# Metatranscriptional Response of Chemoautotrophic *Ifremeria nautilei* Endosymbionts to Differing Sulfur Regimes

**DOI:** 10.3389/fmicb.2016.01074

**Published:** 2016-07-19

**Authors:** Sherry L. Seston, Roxanne A. Beinart, Neha Sarode, Abigail C. Shockey, Piyush Ranjan, Sangita Ganesh, Peter R. Girguis, Frank J. Stewart

**Affiliations:** ^1^Department of Biology, Alverno CollegeMilwaukee, WI, USA; ^2^Department of Organismic and Evolutionary Biology, Harvard UniversityCambridge, MA, USA; ^3^School of Biology, Georgia Institute of TechnologyAtlanta, GA, USA; ^4^Department of Medical Microbiology and Immunology, University of Wisconsin-MadisonMadison, WI, USA

**Keywords:** *Ifremeria nautilei*, chemoautotroph, endosymbiont, methanotrophic bacteria, Sulfur oxidizers, metatranscriptomics, deep sea vents

## Abstract

Endosymbioses between animals and chemoautotrophic bacteria are ubiquitous at hydrothermal vents. These environments are distinguished by high physico-chemical variability, yet we know little about how these symbioses respond to environmental fluctuations. We therefore examined how the γ-proteobacterial symbionts of the vent snail *Ifremeria nautilei* respond to changes in sulfur geochemistry. Via shipboard high-pressure incubations, we subjected snails to 105 μM hydrogen sulfide (LS), 350 μM hydrogen sulfide (HS), 300 μM thiosulfate (TS) and seawater without any added inorganic electron donor (ND). While transcript levels of sulfur oxidation genes were largely consistent across treatments, HS and TS treatments stimulated genes for denitrification, nitrogen assimilation, and CO_2_ fixation, coincident with previously reported enhanced rates of inorganic carbon incorporation and sulfur oxidation in these treatments. Transcripts for genes mediating oxidative damage were enriched in the ND and LS treatments, potentially due to a reduction in O_2_ scavenging when electron donors were scarce. Oxidative TCA cycle gene transcripts were also more abundant in ND and LS treatments, suggesting that *I. nautilei* symbionts may be mixotrophic when inorganic electron donors are limiting. These data reveal the extent to which *I. nautilei* symbionts respond to changes in sulfur concentration and species, and, interpreted alongside coupled biochemical metabolic rates, identify gene targets whose expression patterns may be predictive of holobiont physiology in environmental samples.

## Introduction

Hydrothermal vents are dynamic ecosystems where the vigorous emission of hot, chemically reduced fluid from the seafloor into the surrounding seawater results in a temporally and spatially variable physico-chemical environment. The dominant animals in these habitats live in partnership with bacterial symbionts that oxidize chemical reductants from the venting fluid, including hydrogen sulfide, methane, and hydrogen (Tivey, 2007), as well as thiosulfate, an intermediate oxidation product of sulfide that accumulates at some vents (Gru et al., 1998; Waite et al., 2008; Gartman et al., 2011). The symbionts oxidize these reduced compounds to fuel carbon fixation (chemoautotrophy), which provides primary nutrition for themselves and their animal hosts (Cavanaugh et al., 2006; Dubilier et al., 2008).

To acquire the chemical substrates needed for symbiont metabolism, symbiotic vent animals live where they can access both emitted fluid and oxygenated seawater (Stewart et al., 2005). Vent symbioses thrive in mixing zones where fluid turbulence causes conditions to change rapidly over small spatial scales such that adjacent animals may experience vastly different chemical and physical environments (Johnson et al., 1986). Sulfur chemistry in these zones can be particularly dynamic, with concentrations of hydrogen sulfide and derivatives of sulfide oxidation (e.g., thiosulfate) varying temporally and in proximity to vent sites (Gru et al., 1998; Mullaugh et al., 2008; Waite et al., 2008; Gartman et al., 2011). Furthermore, venting can halt, either transiently or permanently, due to changes in volcanic activity or the path of fluid flow (Butterfield et al., 1997). The dynamic nature of hydrothermal vents thus exposes organisms to fluctuating concentrations of diverse reductants and oxidants (Shank et al., 1998), and may drive variation in the metabolism and growth dynamics of symbiotic partners (Duperron et al., 2007; Dubilier et al., 2008; Robidart et al., 2011).

Descriptive and experimental studies over the past 30 years have enabled a broad understanding of the chemoautotrophic metabolism of vent symbionts. Physiological experiments with intact animal hosts (e.g., Childress et al., 1986, 1991; Girguis et al., 2000; Girguis and Childress, 2006; Nyholm et al., 2008; Petersen et al., 2011; Goffredi et al., 2014; Beinart et al., 2015) and excised symbionts (Belkin et al., 1986; Fisher et al., 1987; Wilmot and Vetter, 1990; Childress et al., 1991; Nelson et al., 1995) have established that vent symbionts can fix inorganic carbon via the oxidation of hydrogen sulfide, thiosulfate, hydrogen and/or methane. Separately, analyses of symbiont gene content (Kuwahara et al., 2007; Newton et al., 2007; Robidart et al., 2008; Nakagawa et al., 2014), gene and protein expression (Markert et al., 2007, 2011; Nyholm et al., 2008; Robidart et al., 2011; Gardebrecht et al., 2012; Wendeberg et al., 2012; Sanders et al., 2013), and enzyme activity (Felbeck, 1981; Stein et al., 1988; Robinson et al., 1998) from vent symbioses have clarified the pathways that symbionts employ for these metabolisms.

While it has been speculated that shifts in chemical availability are coupled to rapid physiological responses by the host, the symbiont, or both (Girguis and Childress, 2006), inferences made from freshly collected animals reveal at best a general picture of the conditions experienced *in situ*, making it challenging to identify the relationships between environmental conditions, symbiont/host physiology, and shifts in the metabolic coupling of the symbiont and host. A greater understanding of how the environment shapes the physiology of vent symbionts requires controlled experiments that couple measurements of metabolic rates with analyses of gene expression. Accordingly, we conducted high-pressure respirometric experiments on the symbiotic vent snail *Ifremeria nautilei* to characterize changes in symbiont gene expression (metatranscriptomes) under varying regimes of reduced sulfur species and availability.

*I. nautilei* lives in mixing zones around hydrothermal vents in the southwestern Pacific, deriving its nutrition from intracellular gill symbionts that can oxidize both sulfide and thiosulfate to fuel autotrophy (thioautotrophy; Beinart et al., 2015). Previous characterization of the *I. nautilei* gill symbiont community revealed associations with a lineage of γ-proteobacterial sulfur oxidizers from the Order Chromatiales (Urakawa et al., 2005; Suzuki et al., 2006). Methanotrophic bacteria may also be present (Gal'chenko et al., 1992; Borowski et al., 2002), although their representation across host populations is inconsistent (Windoffer and Giere, 1997; Suzuki et al., 2006) and molecular characterization of *I. nautilei* methanotrophs is lacking (Petersen and Dubilier, 2009). We herein present 16S rRNA gene and metatranscriptome data elucidating symbiont community composition and metabolism over a range of environmentally relevant conditions: (i) no electron donor (ND), (ii) 105 μM hydrogen sulfide (LS), (iii) 350 μM hydrogen sulfide (HS), and (iv) 300 μM thiosulfate (TS). These experiments clarify pathways of sulfur, nitrogen, and carbon metabolism in the *Ifremeria*-associated bacterial community and provide the first molecular evidence for the activity of methanotrophic bacteria in the gill-associated community. The results are derived from experiments designed to study the effects of geochemical dynamics on sulfur oxidation and carbon fixation rates (Beinart et al., 2015). An analysis of symbiont transcripts in light of the observed metabolic rates reveals how *I. nautilei* symbionts sustain chemoautotrophic activity at low sulfide concentrations, but exhibit a marked change in their physiology as sulfur availability increases.

## Materials and methods

### Animal collection and experimental incubations

As described in Beinart et al. (2015) *I. nautilei* were collected and incubated in four separate experiments that were performed to compare symbiont metabolism and gene expression when exposed to 105 μM hydrogen sulfide (low sulfide, LS), 300 hydrogen sulfide μM (high sulfide, HS), 300 thiosulfate μM (TS), or no exogenous electron donor (no donor, ND). Snails were collected from the vent fields ABE (HS and TS) or Tu'i Malila (LS and ND) at the Eastern Lau Spreading Center by the remotely operated vehicle JASON II during expedition TM-235 in 2009 (Table 1). Snails were brought to the ship in insulated containers and kept in 4°C seawater. Individuals responsive to the touch were either immediately preserved as described below or were transferred to flow-through, 2.68 L titanium aquaria in a high-pressure respirometry system (HPRS) that maintains the animals at 3000 psi. All treatments had five snails per aquaria, except the LS treatment, which had only four individuals. Snails in the HPRS were acclimated for 8 h prior to the start of the treatment incubations, during which time snails were exposed to input water comprised of filtered seawater amended with sodium nitrate (NaNO_3_, final concentration 40 μM) bubbled with carbon dioxide, oxygen, and nitrogen to achieve concentrations of 4 mM, >300 μM, and 400 μM, respectively. Snails used for the HS, TS, and LS treatments were acclimated without an electron donor, whereas those used in the ND treatment were acclimated with 300 μM sulfide, achieved by bubbling input water with gaseous hydrogen sulfide (5% H_2_S/95% N_2_). The addition of sulfide during ND snail acclimation was done to avoid overly stressed or starved individuals at the end of the long experimental period. Following acclimation, input water for TS, HS, and LS treatments were amended with either 300 μM sodium thiosulfate (NaS_2_O_3_) or bubbled with gaseous hydrogen sulfide (5% H_2_S/95% N_2_) to achieve target concentrations, whereas ND treatments received no energy source. For the duration of each experiment (between 27 and 40 h, Table 2), the concentration of hydrogen sulfide, thiosulfate, and polysulfide was monitored in the input water and effluent from the aquaria via an inline voltammetric microelectrode, allowing for the calculation of mass-specific rates of sulfur metabolism. Oxygen (O_2_) was detectable in the outflow of all vessels during the experiments, as measured by an oxygen optode (Golden Scientific), however, we were unable to quantify exact oxygen concentrations due to problems with calibration. Though the total length of each experiment varied due to logistical constraints, the sulfur metabolism and/or respiration of the animals in all experiments reached steady state after at least 10 h into the experiment. In addition, the incorporation of inorganic carbon (i.e., carbon fixation) by snail symbionts was measured by amending input seawater with isotopically labeled sodium carbonate (Na13CO3; 99.9% atom percent; Icon Services). A full description of the experimental system, conditions, measurements, and analysis can be found in Beinart et al. (2015).

**Table 1:** **Date and location of experimental animal collection**.

**Treatment**	**Vent Field**	**Dive**	**Date**	**Lat**	**Lon**	**Depth (m)**
HS	ABE	J2-423	May 22, 2009	−20° 45.794′	−176° 11.466′	2152
TS	ABE	J2-427	May 27, 2009	−20° 45.797′	−176° 11.477′	2150
ND	Tu'i Malila	J2-428	May 29, 2009	−21° 59.358′	−176° 34.086′	1885
LS	Tu'i Malila	J2-430	June 2, 2009	−21° 59.354′	−176° 34.080′	1885

**Table 2:** **Net sulfur uptake (oxidation) and carbon incorporation by ***Ifremeria*** during experiments**.

	**Experiment duration (h)**	Input sulfur concentration (μM)^*^	**Oxidation Rate (μmoles g^−1^ h^−1^)**	**Individual**	**Gill tissue δ^13^C**	**Rate C_inc_ (μmoles g^−1^ h^−1^)**
HS	34	349 (329, 387)	7.2 ± 0.75	1	−29.74	0.00
				2	1.41	1.64
				3	217.29	9.57
TS	37	276 (216, 310)	12.0 ± 2.1	1	15.16	4.22
				2	87.93	8.67
				3	46.02	7.06
ND^**^	34	BDL	NA	1	−31.40	0.00
				2	−30.82	0.00
				3	−31.16	0.00
LS	40	105 (57, 137)	2.8 ± 0.77	1	−28.10	0.00
				2	−25.65	0.21
				3	2.85	0.71

*Average (min, max) concentrations of sulfur compounds in input water as measured via a voltammetric electrode and/or with Klein method. Wet mass-specific sulfur oxidation rates are shown as the average ± S.D. Incorporation of stable isotopic label shown as per mil values of experimental I. nautilei gill tissue and wet mass-specific rates of incorporation into gill tissue (Beinart et al., 2015)*.

* *Detection limits: Sulfide 0.20 μM; Thiosulfate, 30 μM*.

** *Sulfur compounds were not added. Below detection limit (BDL) for all measurable sulfur compounds in both input and effluent*.

### Nucleic acid extraction

Immediately after collection from the vent field or at the conclusion of each experiment, animals were confirmed to be alive via responsiveness to touch, and then quickly excised from their shells. Subsamples of gill tissue from each individual were promptly homogenized with a Tissue Tearor and preserved in Trizol™ (LifeTechnologies) and stored at −80°C. RNA was extracted via the manufacturer's protocol. DNA from the same samples was back-extracted from the resulting interphase with a buffer consisting of 4 M guanine thiocyanate, 50 mM sodium citrate, and 1 M Tris (free base). The DNA was then extracted again with chloroform isoamyl alcohol and precipitated with isopropanol. The resulting DNA pellets were washed twice with 75% ethanol and air-dried. DNA pellets were resuspended in 8 mM sodium hydroxide, adjusted to pH 7–8 with 0.1 M HEPES and amended with 1 mM EDTA.

### 16S rRNA gene amplification, sequencing, and analysis

High-throughput sequencing of dual-indexed PCR amplicons encompassing the V4 hypervariable region of the 16S rRNA gene was used to assess the identity and relative abundance of symbionts in *I. nautilei* gill tissue. Amplicons were synthesized using Platinum PCR supermix (Life Technologies) with V4-specific primers F515 and R806 (Caporaso et al., 2010). Both forward and reverse primers were barcoded and appended with Illumina-specific adapters as per instructions by Kozich et al. (2013). Thermal cycling conditions were: initial denaturation at 94°C (3 min), followed by 30 cycles of denaturation at 94°C (45 s), primer annealing at 55°C (45 s), and primer extension at 72°C (90 s), followed by final extension at 72°C for 10 min. Amplicons were analyzed by agarose gel electrophoresis to verify size (~400 bp) and purified using Diffinity RapidTip2 PCR purification tips. Barcoded and Illumina adaptor-appended amplicons for each sample were pooled at equimolar concentrations and sequenced on an Illumina MiSeq (software v.2.4.0.4) using a 500 cycle MiSeq reagent kit, with 5% PhiX added to increase sequence diversity.

Demultiplexed amplicon read pairs were quality trimmed with Trim Galore! (Babraham Bioinformatics), using a base Phred33 score threshold of Q25 and a minimum length cutoff of 100 bp. High quality paired reads were then merged using the software FLASH (Magoč and Salzberg, 2011). Merged reads were analyzed using the software pipeline QIIME v1.8.0 (Caporaso et al., 2010). Reads were first screened for chimeras using QIIME's identify_chimeric_seqs.py script with usearch61. Non-chimeric sequences were clustered into Operational Taxonomic Units (OTUs) at 97% sequence similarity using the open reference OTU picking protocol with the script pick_open_reference_otus.py. Taxonomy was assigned to representative OTUs from each cluster using the Greengenes reference database (Aug 2013 release; DeSantis et al., 2006). The core set of QIIME diversity analyses was performed using the workflow script core_diversity_analyses.py at an even sampling depth of 19 978 sequences. One representative sequence from the 5 most abundant OTUs was aligned with related proteobacterial sequences using MUSCLE in MEGA 5.2.2 (Edgar, 2004). Phylogenetic analysis of the aligned representative sequences for each OTU was performed to further clarify the identity of host-associated 16S rRNA gene sequences. A maximum-likelihood tree was constructed using 252 unambiguously aligned nucleotide positions in webPRANK (Löytynoja and Goldman, 2010), and included sequences from diverse chemoautotrophic symbionts and free-living marine bacteria found in Genbank. Bootstrap analysis (1000 replicates) was performed to assess node confidence.

### Metatranscriptome library preparation, sequencing, and analysis

Analysis of protein-coding genes and transcripts followed that of Stewart et al. (2012). Aliquots (50 ng) of total RNA were used to prepare cDNA libraries using the ScriptSeq™ v2 RNA-Seq Library Preparation Kit (Epicenter, Madison, WI, USA) according to manufacturer instructions. Briefly, cDNA was synthesized from fragmented total RNA (rRNA was not removed) using reverse transcriptase and amplified and barcoded using ScriptSeq Index PCR Primers (Epicenter) to generate single-indexed cDNA libraries. cDNA libraries were pooled and sequenced on an Illumina MiSeq using a 500 cycle kit. Barcoded sequences were de-multiplexed and filtered to remove low-quality reads (Phred score <25). Paired end sequences were merged using custom scripts incorporating the FASTX toolkit (http://hannonlab.cshl.edu/fastx_toolkit/index.html) and USEARCH algorithm (Edgar, 2010), with criteria of minimum 10% overlap and 95% nucleotide identity within the overlapping region

Prokaryotic and eukaryotic ribosomal RNA (rRNA) reads were separated from total RNA reads with riboPicker (Schmieder et al., 2012) using the comprehensive non-redundant rRNA database (2012 update) as reference. Reads with at least 75% nucleotide identity and 50% query coverage were considered a match to a ribosomal sequence. Reads identified as rRNA were submitted to MG-RAST (Meyer et al., 2008) to analyze overall rRNA taxonomic distribution. A subset of 100 000 rRNA reads from each sample were also analyzed in QIIME v 1.8 as described above.

Non-rRNA reads from each sample were processed with PRINSEQ Lite 0.20.4 (Schmieder and Edwards, 2011) to remove exact duplicate sequences (reads with 100% identity and equal length). Non-rRNA reads were annotated using BLASTX against the NCBI-nr database of non-redundant protein sequences (as of November 2013). BLASTX results were examined in MEGAN5 (build 4.1; Huson et al., 2011) and matches to bacterial and archaeal genes above a bit score of 50 were retained and classified according to functional categories based on the SEED subsystems ontology (Overbeek et al., 2005). Reads annotated as unassigned or eukaryotic were not included in further analyses. Each metatranscriptome was normalized to the sample with the smallest number of bacterial non-rRNA reads (Snail TS-1: 11 654 reads) in MEGAN5 by randomly subsampling 11 654 reads from each metatranscriptome 1000 times. The average count for each SEED subsystem category rounded to the nearest integer was used in all subsequent analyses. Differential expression of SEED Subsystem categories and proteins was analyzed with the baySeq R package v 2.6 (Hardcastle and Kelly, 2010). The heat map and distance clustering of SEED subsystems and samples was computed using heatmap.2 within the gplots R package (Warnes et al., 2013). To validate the results obtained in MEGAN and explore expression of particular genes of interest, BLASTX results (bit score > 50) were manually examined via keyword searches based on NCBI-nr annotations independent of MEGAN, as in (Canfield et al., 2010). Manual searches focused on enzymes of the Calvin Benson Bassham (CBB) cycle, reductive tricarboxylic acid (rTCA) cycle, methane oxidation, and nitrogen and sulfur metabolism in bacteria (Klotz and Stein, 2008) including: pyrophosphate-dependent phosphofructokinase, membrane-bound proton-translocating pyrophosphatase, ATP citrate lyase, 2-oxoglutarate oxidoreductase, fumarate reductase, SoxABCDHWXYZ proteins, Sulfide:quinone (oxido)reductase (Sqr), sulfide dehydrogenase (Fcc), APS reductase (AprAB), sulfate adenylytransferase (Sat), DsrABCEFHJKLMOPNR proteins, heterodisulfide reductase, methane monooxygenase, methanol dehydrogenase, pyrroloquinoline quinone synthase, 3-hexulose-6-phosphate synthase, and 3-hexulose-6-phosphate isomerase.

*Amplicon and metatranscriptome sequences are available through NCBI under BioProject ID PRJNA288684*.

## Results and discussion

### Metabolic activity

*Ifremeria nautilei* individuals were collected in four separate dives from four locations within the ABE and Tu'i Malila vent fields of the Eastern Lau Spreading Center (Table 1 and Figure S1). All individuals were subjected to an 8-h acclimation period in pressurized aquaria before the treatment incubations. Snails used for the HS, TS, and LS treatments were acclimated without an electron donor, whereas those used in the ND treatment were acclimated with 300 μM sulfide. After acclimation, treatment conditions were established in the aquaria and remained constant for 27–40 h. The same snails that were used for rate measurements were used for subsequent symbiont community composition and metatranscriptome analysis. Sulfur oxidation and carbon fixation rates for all individuals were reported in Beinart et al. (2015) and are summarized here for convenience in interpreting the gene expression patterns. Sulfur metabolism rates were measured by assessing the net flux of sulfide, thiosulfate, and polysulfide through high-pressure aquaria under each treatment. *I. nautilei* demonstrated net uptake (oxidation) of sulfide or thiosulfate when these compounds were supplied across all tested conditions (Table 2). Mass-specific sulfur oxidation rates were highest in *I. nautilei* exposed to 300 μM of thiosulfate (TS), with the next highest rates in the HS followed by the LS treatments (Table S2). The measurement of net sulfur flux through the aquaria also revealed the excretion of polysulfides by *I. nautilei* when exposed to the HS treatment (Beinart et al., 2015). Oxygen (O_2_) was detectable in HPRS outflow of all treatments during the experiments, indicating that complete anaerobiosis was not established in any treatment.

Symbiont carbon fixation was observed in treatments exposed to both sulfide and thiosulfate (Table 2). In contrast to sulfur metabolism rates, which were based on the collective uptake by all experimental individuals (i.e., sulfide disappearance from aquaria) and therefore reflected an average metabolic rate across individuals per treatment, carbon fixation rates were measured by the incorporation of stable isotope labeled (^13^C) inorganic carbon into individual snails. Compared to symbiont-free foot tissue (average δ^13^C −28.0 ‰± 0.95 s.d; Beinart et al., 2015), elevated carbon isotopic compositions were detected in the gills of *I. nautilei* individuals exposed to HS, LS, and TS (Table 2), whereas carbon incorporation was not observed in any individuals in the no electron donor (ND) treatment. Although mass-specific incorporation rates were relatively consistent among individuals in the TS treatment, rates differed widely among individuals within the HS and LS treatments. Notably, an individual in the HS treatment demonstrated the highest rate across all treatments, while other HS individuals showed low or undetectable carbon incorporation (Table 2). This pattern, seen also in the LS treatment, reflects the position of the individuals in the aquaria, with the most productive individuals closest to the incoming water, suggesting that metabolism by downstream individuals was limited by the activity of those upstream (Beinart et al., 2015).

### Symbiont taxonomic composition—16S rRNA gene amplicons

**Figure 1:**
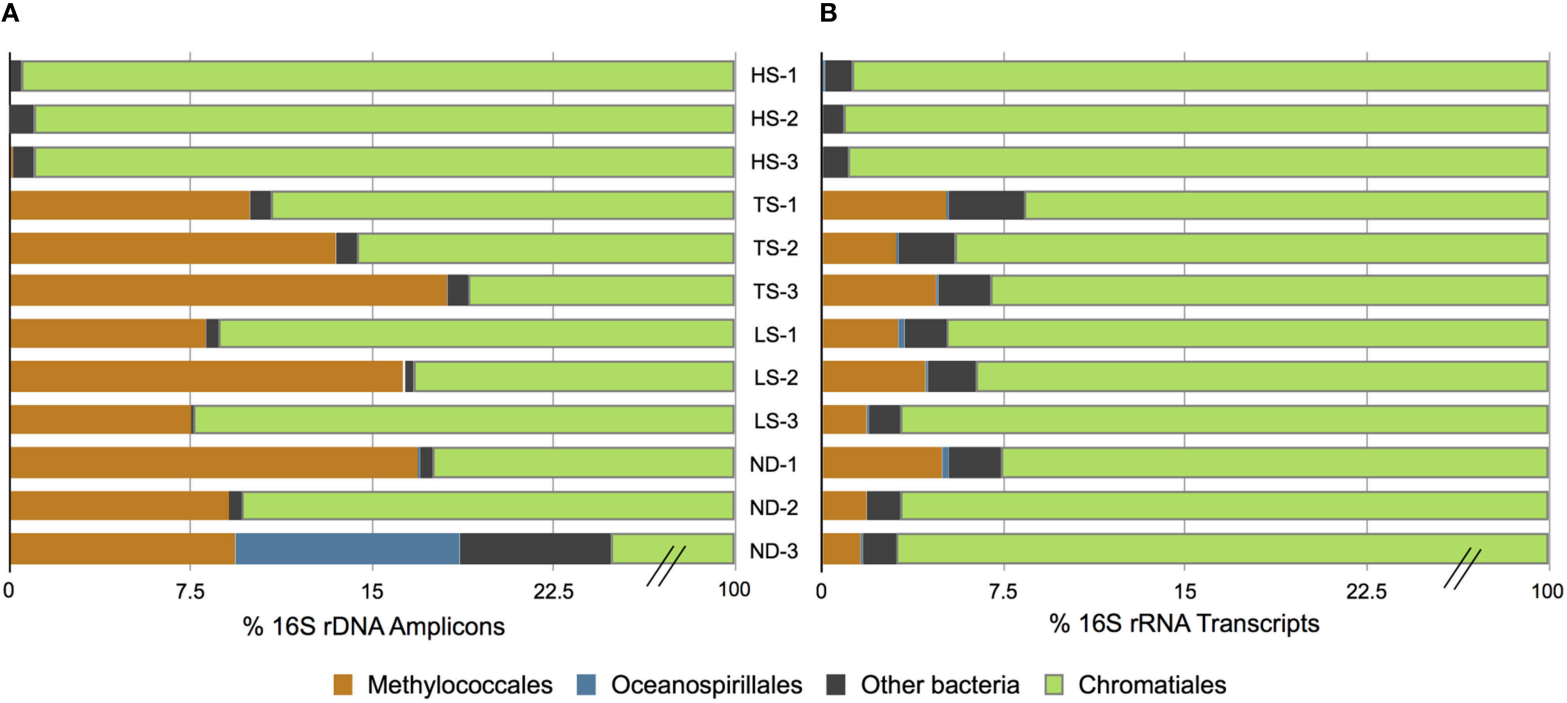
**Distribution of amplified 16S rRNA genes (A) and 16S rRNA transcripts (B) in ***Ifremeria nautilei*** samples from experimental shipboard incubations**.

**Figure 2:**
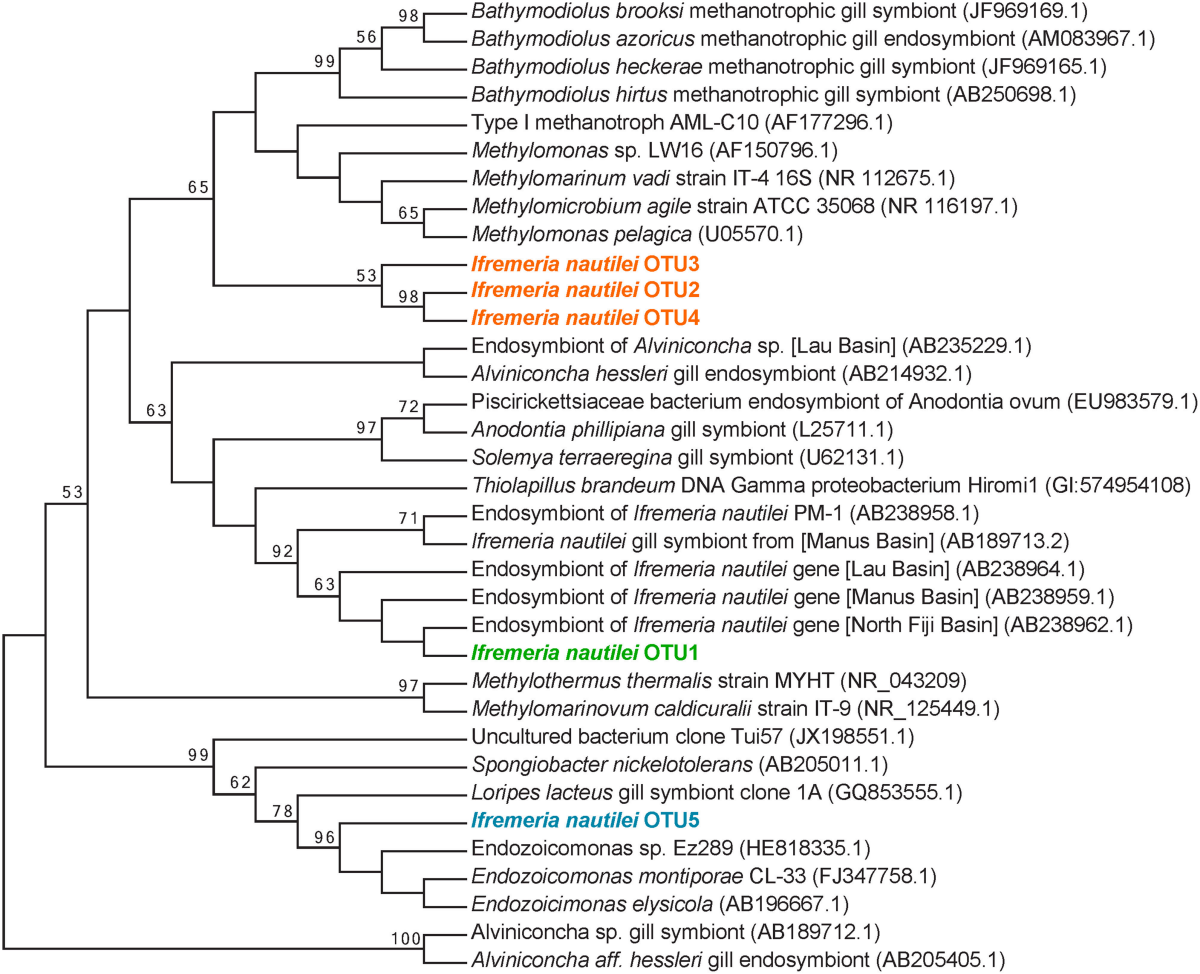
**Maximum-likelihood phylogenetic tree of the top 5 most abundant 16S rRNA gene OTUs**. The 5 OTUs shown here accounted for 98% of all 781 662 reads analyzed. The tree is based on 252 unambiguously aligned nucleotide positions. Two ε-proteobacterial *Alviniconcha hessleri* symbionts are used as the outgroup. The numbers to the left of each node are bootstrap values obtained from 1000 replicates; only values greater than 50% are indicated.

At the conclusion of each incubation, gills of three host individuals were immediately sampled to assess symbiont phylogenetic affiliation and gene transcription profiles. Illumina sequencing of 16S rRNA gene (DNA) amplicons revealed that all 12 experimental *I. nautilei* hosted highly related communities of symbionts (Figure 1). A single OTU (97% similarity cluster) within the order *Chromatiales* of the γ-proteobacteria was dominant across all individuals, accounting for 90.1% of all sequences (Figure 1). A representative sequence from this OTU clustered phylogenetically with the known sulfur-oxidizing *I. nautilei* symbiont, differing from published sequences by 1-2 nucleotides (<0.8%; Figure 2).

Amplicon sequencing also detected putative methanotrophic bacteria in association with *I. nautilei* used in the treatments, although at much lower abundance than the dominant *Chromatiales* symbionts. Sequences related to the *Methylococcaceae, a* γ-proteobacterial family of methanotrophic bacteria, represented 7.7% of all amplicons (all individuals combined) and three distinct OTUs (Figure 2). These OTUs were abundant in the TS, LS, and ND incubations (up to 16.8% of amplicons from a single host), but represented <0.02% of sequences from individuals in the HS incubation (Figure 1, and discussed in more detail below). The *Methylococcaceae* OTUs did not cluster with the methanotrophic symbionts of other animals, but rather formed a separate cluster with a cloned 16S rRNA sequence from a shallow hydrothermal vent system (Hirayama et al., 2007; Figure 2). These findings represent the first published DNA sequences identifying a γ-proteobacterial methanotrophic symbiont in *I. nautilei*. Previous electron microscopy studies have observed multiple bacterial morphotypes in the gill cells of some *I. nautilei* individuals, including a low abundance morphotype with stacked cytoplasmic membranes characteristic of type I methanotrophs (Gal'chenko et al., 1992; Borowski et al., 2002). However, prior molecular analyses detected only the *Chromatiales* symbiont (Windoffer and Giere, 1997; Borowski et al., 2002; Urakawa et al., 2005; Suzuki et al., 2006).

The fifth most abundant OTU recovered from the amplicon library was affiliated with the genus *Endozoicomonas* of the order *Oceanospirillales*, and accounted for 0.5% of all sequences (Figure 2). *Endozoicomonas* bacteria are aerobic heterotrophs that appear commonly in association with marine invertebrates (Dubilier et al., 2008). There is evidence that *Oceanospirillales* are intranuclear parasites that cause host cell death in symbiotic bathymodiolin mussels (Zielinski et al., 2009). Intracellular *Oceanospirillales* have also been observed in the hydrothermal vent gastropod *Alviniconcha* that co-occurs with *I. nautilei* in the Lau Basin, although the nature of the association is not clear (Beinart et al., 2014).

**Figure 3:**
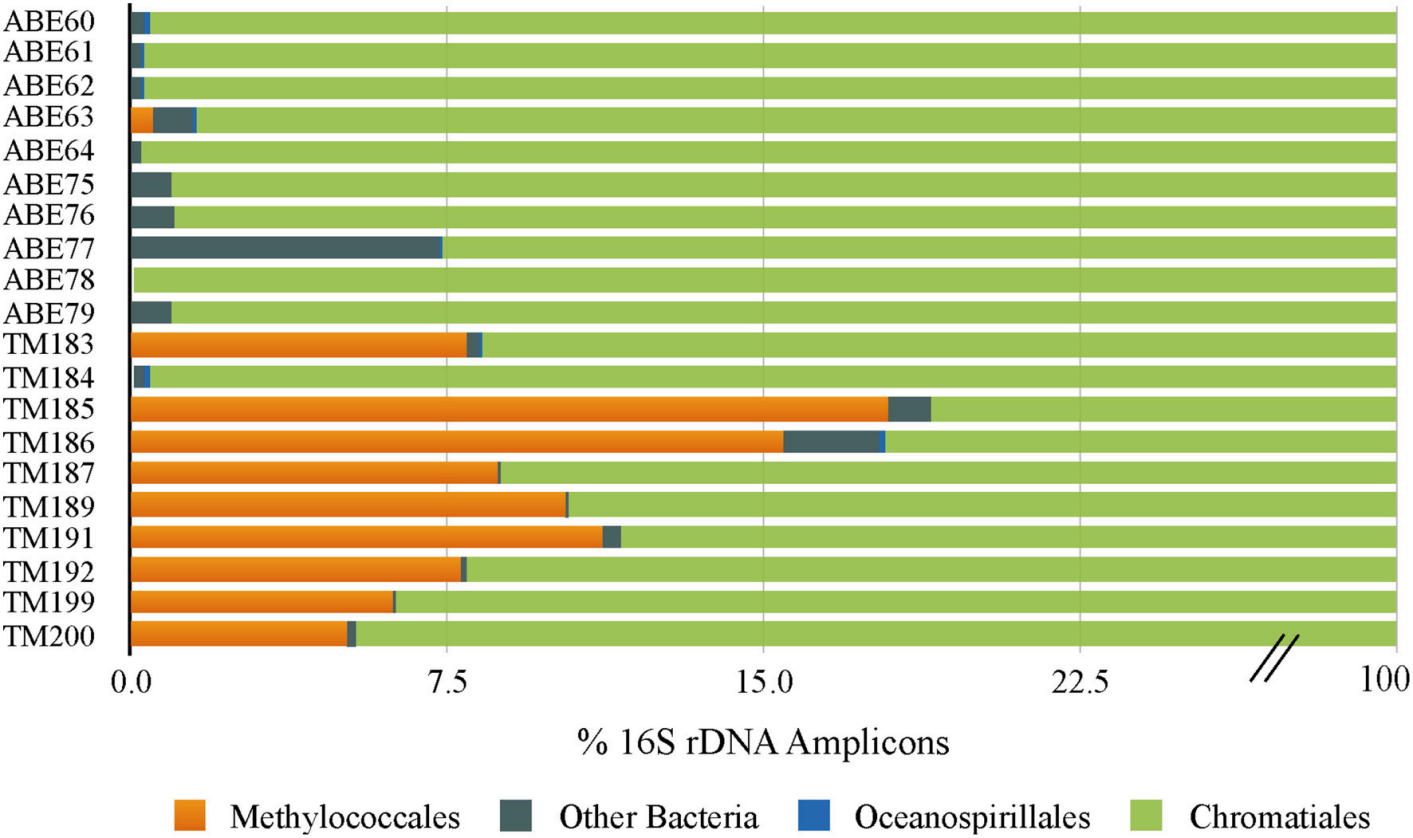
**Bar graph showing the distribution of 16S rRNA OTUs in ***Ifremeria nautilei*** from ABE and Tui Malila vent fields**.

To investigate whether differences in symbiont diversity resulted from incubation conditions or from *in situ* variability among the sampled populations, we extended the 16S rRNA gene analysis to include 10 snails collected from the same vent fields as the experimental animals. This analysis revealed a similar dominance of the single *Chromatiales* OTU, but also considerable variation in the relative abundance of *Methylococcaceae* among host individuals (Figure 3). Notably, *Methylococcaceae* OTUs composed < 0.05% of the symbiont population in snails from the ABE vent field collected during the same dive as snails HS1-3 used in the HS treatment, while the symbiont population within snails from the Tu'i Malila vent field contained 5–17% *Methylococcaceae*. These results suggest that the low proportion of *Methylococcaceae* in the HS 16S rRNA gene datasets may reflect environmental variation among symbiont populations rather than an effect of incubation conditions (Figure 1). Our results, from both experimental and environmental specimens, highlight the potential for local variation in the proportional abundances of *Chromatiales* and *Methylococcaceae* symbionts in *I. nautilei*. Additional studies are necessary to confirm whether such variation is linked with environmental sulfide and methane availability, as shown for other dual chemosynthetic symbioses (Duperron et al., 2011).

### Metatranscriptome characteristics and taxonomic composition

Each metatranscriptome was derived from a single snail and consisted of 1 to 5 million high quality paired sequence reads representing both symbiont and host RNA (Table 3). Metatranscriptomes were generated from the same 12 individuals (3 from each treatment) that were used in the metabolic experiments and phylogenetic analyses. Of the reads generated, 69–77% represented rRNA, while 5–9% represented mRNA transcripts with matches to protein-coding genes in the NCBI nr database. The remaining 16–24% of the reads not identified as either rRNA or mRNA may represent unidentified protein-coding genes or enzymatic and regulatory RNA. Approximately 51–73% of mRNA transcripts identified in MEGAN were eukaryotic in origin, with only 8–12% of these transcripts successfully assigned a functional annotation. The single most abundant transcript identified in all 12 metatranscriptomes (2–7% of all identified protein-coding transcripts) matched the cytochrome oxidase subunit I gene of *I. nautilei* with > 99% identity, confirming the species identity of the host individuals. Other abundant eukaryotic transcripts identified in all host individuals regardless of treatment included cytochrome oxidase subunit II, tRNAs, NADH dehydrogenase complex I, fumarate reductase, cytochrome bc1 complex, and ATP synthase subunits, suggesting that all snails were metabolically active at the time of sampling and aerobic conditions were maintained throughout all experiments.

**Table 3:** **Metatranscriptome sequence characteristics**.

**Snail**	**Total reads**	**rRNA reads**	**Non-rRNA reads**	**BLASTX Hits**	**MEGAN assigned proteins**	**Eukaryotic assigned proteins**	**Bacterial assigned proteins**	**Other assigned proteins^a^**
HS-1	2,106,871	1,500,965	605,906 (28%)	175,997	114,267	74,424 (65%)	36,481 (32%)	3,362 (3%)
HS-2	3,097,455	2,399,502	697,953 (23%)	215,535	150,694	87,022 (58%)	56,710 (38%)	6,962 (4%)
HS-3	2,753,601	2,074,095	679,506 (25%)	226,213	156,536	79,203 (51%)	75,514 (48%)	1,819 (1%)
TS-1	1,138,408	870,307	268,101 (24%)	63,447	42,524	29,811 (70%)	11,654 (27%)	1,059 (3%)
TS-2	3,140,132	2,412,371	727,761 (23%)	192,802	138,917	85,890 (62%)	48,067 (35%)	4,960 (3%)
TS-3	2,903,835	2,219,159	684,676 (24%)	188,561	138,659	83,649 (60%)	50,116 (36%)	4,894 (4%)
LS-1	1,280,435	974,592	305,843 (24%)	69,872	42,796	29,272 (68%)	13,051 (30%)	473 (2%)
LS-2	3,511,480	2,583,832	927,648 (26%)	265,621	184,359	109,752 (60%)	67,796 (37%)	6,811 (3%)
LS-3	2,958,987	2,295,224	663,763 (29%)	189,656	138,931	92,533 (67%)	41,765 (30%)	4,633 (3%)
ND-1	2,115,702	1,462,295	653,407 (31%)	153,995	96,528	70,884 (73%)	23,290 (24%)	2,354 (3%)
ND-2	4,982,423	3,854,530	1,127,887 (23%)	318,447	229,893	128,470 (56%)	88,102 (38%)	13,321 (6%)
ND-3	3,775,724	2,717,615	1,058,109 (28%)	298,774	203,180	137,423 (68%)	59,246 (29%)	6,511 (3%)

a *Includes proteins predicted to be of viral, archaeal, or unknown origin*.

Bacterial 16S rRNA transcripts composed 19–53% of the total small subunit (SSU) rRNA reads (16S + host 18S) in each metatranscriptome. Interestingly, 16S rRNA represented a higher proportion of total SSU transcripts in metatranscriptomes from the HS treatments (average 45 ± 7%) compared to those from the other treatments (average 26 ± 6%; Table 3). This difference may reflect variation in either symbiont density or activity. This is consistent with the aforementioned metabolic data, and suggests that at elevated sulfide concentrations the bacterial symbiont population is more active. The vast majority of 16S rRNA transcripts (91.6–99.1% in all metatranscriptomes) matched sequences related to the *Chromatiales* (Figure 1). Consistent with the 16S gene amplicon results, a proportion of 16S rRNA transcripts (1.6–5.1%) from the TS, LS, and ND metatranscriptomes were classified as *Methylococcaceae*, whereas *Methylococcaceae* represented < 0.02% of total 16S transcripts in the three HS transcriptomes (Figure 1). In all specimens, the proportion of *Methylococcaceae* sequences in the 16S rRNA datasets derived from metatranscriptomes was approximately 1/4 the proportion observed in the DNA-amplified 16S gene datasets. This consistently lower proportion in the transcript data could indicate a low cellular RNA to DNA ratio, suggesting that *Methylococcaceae* bacteria were less metabolically active than the sulfur-oxidizing population during the incubations. This is consistent with the assumption that all of the incubations lacked appreciable methane or other C1 compounds that methylotrophs typically metabolize (neither methane nor any other C1 compounds were added to these incubations). Alternatively, the discrepancy between the DNA and RNA data could result from PCR bias and uneven amplification of different templates. Finally, consistent with the amplicon data, a minor fraction (0.2–0.5%) of 16S rRNA transcripts in each metatranscriptome matched sequences of the *Oceanospirillales* order (Figure 1).

### Differential gene expression

To detect differential expression, protein-coding transcripts were assigned to functional SEED subsystems (Level 1 and 2) and proteins (Level 4) in MEGAN5 (Huson et al., 2011). The baySeq R package was then used to determine which model (treatment grouping) best explained subsystem and protein expression patterns (Table 4). Differential expression patterns were best explained by dividing the metatranscriptomes into two groups based on the concentration of electron donor in the incubation experiments. Grouping transcriptomes according to high electron donor (HD: HS and TS treatments) vs. low electron donor (LD: LS and ND treatments) conditions explained an estimated 39% of Level 1 subsystem differential expression, compared to 24% when transcriptomes were grouped according to the four individual treatments (Table 4). This trend was also observed when transcripts were assigned to Level 3 SEED subsystems and functional proteins (Table 4 and Tables S1, S2). Other models, including one comparing the thiosulfate treatment to the sulfide-containing treatments, explained less than 20% of variation at all subsystem levels. The availability of electron donor, and not donor compound type, therefore appears to be the strongest driver of symbiont differential gene expression in these experiments.

**Table 4:** **Estimated proportion of differential expression based on posterior likelihoods of differential expression among treatments**.

	**Estimated proportion of differential expression**		
**Model**	**SEED level 1 (28 subsystems)**	**SEED level 3 (344 subsystems)**	**SEED level 4 (1086 genes)**
(HS,HS,HS,TS,TS,TS) (LS,LS,LS,ND,ND,ND)	0.39 (8)^*^	0.29 (23)	0.49 (62)
(HS,HS,HS) (TS,TS,TS) (LS,LS,LS) (ND,ND,ND)	0.24 (6)	0.05 (10)	0.04 (2)
(HS,HS,HS,TS,TS,TS,LS,LS,LS) (ND,ND,ND)	0.18 (1)	0.07 (0)	0.16 (0)
(HS,HS,HS,LS,LS,LS) (TS,TS,TS)	0.10 (0)	0.09 (0)	0.21 (1)

*HS, High Sulfide; TS, Thiosulfate; LS, Low Sulfide; ND, No e- donor*.

* *Number of subsystems differentially expressed (FDR = 0.05) shown in parentheses*.

While our data indicate electron donor availability as the major driver of symbiont transcriptional variation, it is possible that factors independent of treatment conditions also affected the observed transcription patterns. First, some genes may be constitutively transcribed at uniform levels among treatments. Second, both the host and symbionts may produce storage molecules that could—over the duration of the incubations—dampen the transcriptional and physiological response to our treatments. For example, some chemoautotrophic symbionts produce elemental sulfur that can be stored and may be used as energy to drive carbon fixation when exogenous reductants are absent or low (Vetter, 1985; Wilmot and Vetter, 1990; Windoffer and Giere, 1997; Vetter and Fry, 1998; Pflugfelder et al., 2005). The presence of such molecules could enable symbionts to maintain thioautotrophic growth and associated gene expression patterns without exogenous reduced sulfur. It is possible that the snails used here may have contained varying amounts of storage compounds due to differences in sulfide availability at the collection sites. Finally, the half-life of mRNA varies widely, generally from minutes to hours (Rauhut and Klug, 1999; Deana and Belasco, 2005), raising the possibility of a decoupling between transcript profiles and the inferred physiological outcomes linked to transcribed genes. However, the long duration during which snails were maintained in HPRS (8 h acclimation + 27–40 h incubation) makes it unlikely that our results reflect transcriptional variation due to differences in *in situ* conditions at the collection sites.

**Figure 4:**
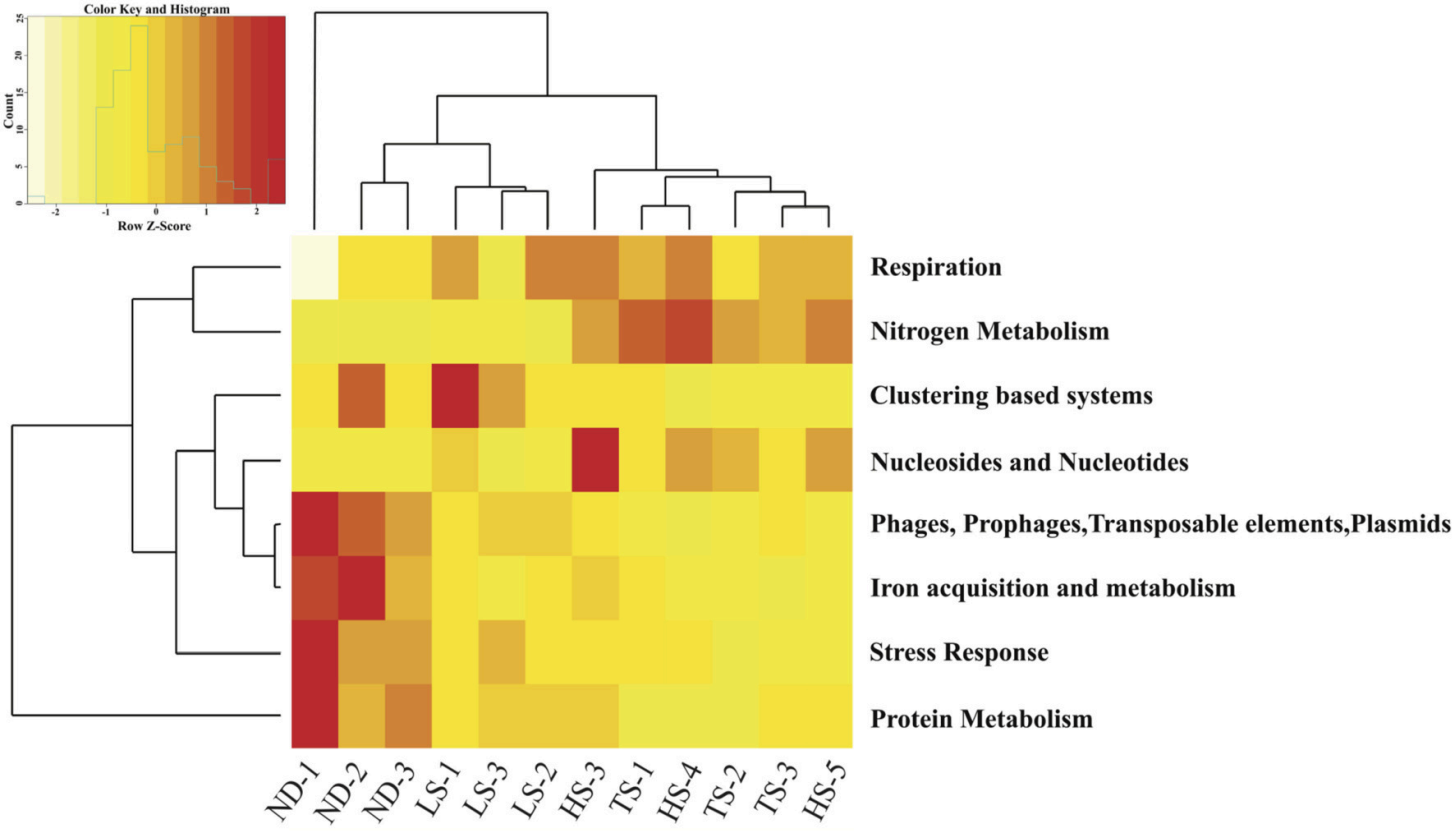
**Heat map displaying hierarchical distance clustering of the eight SEED Subsystems differential expressed under HD vs. LD conditions based on normalized transcript abundance**. Each row corresponds to a SEED subsystem, and each column to an individual experimental *Ifremeria nautilei*. Relative expression levels are indicated by colors shown in the scale at the top left. The dendrogram at the top shows the clustering of the individual snail samples. The dendrogram at left demonstrates the clustering of the SEED Subsystems.

Eight of the 28 Level 1 SEED subsystems differed significantly in expression (FDR < 0.05, Table 4 and Table S1). Sample clustering based on transcript representation in these 8 categories grouped high electron donor (HD) samples separate from low electron donor (LD) samples (Figure 4). In agreement with the higher carbon fixation rates measured directly in HD samples, transcripts associated with nitrogen metabolism, respiration, and nucleosides/nucleotides were more abundant in metatranscriptomes from HD treatments. Closer inspection of differentially expressed genes in these categories indicated that the availability of reduced sulfur compounds increased the representation of transcripts involved in nitrogen assimilation, ATP synthesis, cytochrome oxidase biogenesis, and dissimilatory nitrate reduction (Table S2). In contrast, LD incubations were enriched in transcripts involved in stress response, protein metabolism, phages/prophages/transposable elements/plasmids, clustering-based subsystems, and iron acquisition/metabolism (Figure 4).

It should be noted that many proteins are listed in multiple SEED subsystems. For example, the chaperone protein DnaK is found in both protein metabolism and stress response subsystems. Other proteins are listed in seemingly unrelated subsystems, as is the case for the Fe-S cluster scaffold protein SufB, which is included in the phages/prophages/transposable elements/plasmids subsystem. Thus, drawing definitive conclusions from Level 1 SEED subsystem expression is difficult. Below, we use the results of the differential expression analysis of SEED functional proteins combined with supplemental manual queries of BLASTX results to describe in detail the expression patterns for key processes of symbiont physiology, especially those identified as differentially expressed in baySeq. Based on the 16S rRNA gene and transcript patterns described above, the vast majority of bacterial transcripts described are likely derived from the sulfur-oxidizing symbiont population. However, in the absence of genome data, we are not able to definitively localize all transcripts to a particular symbiont phylotype.

### S metabolism

**Figure 5:**
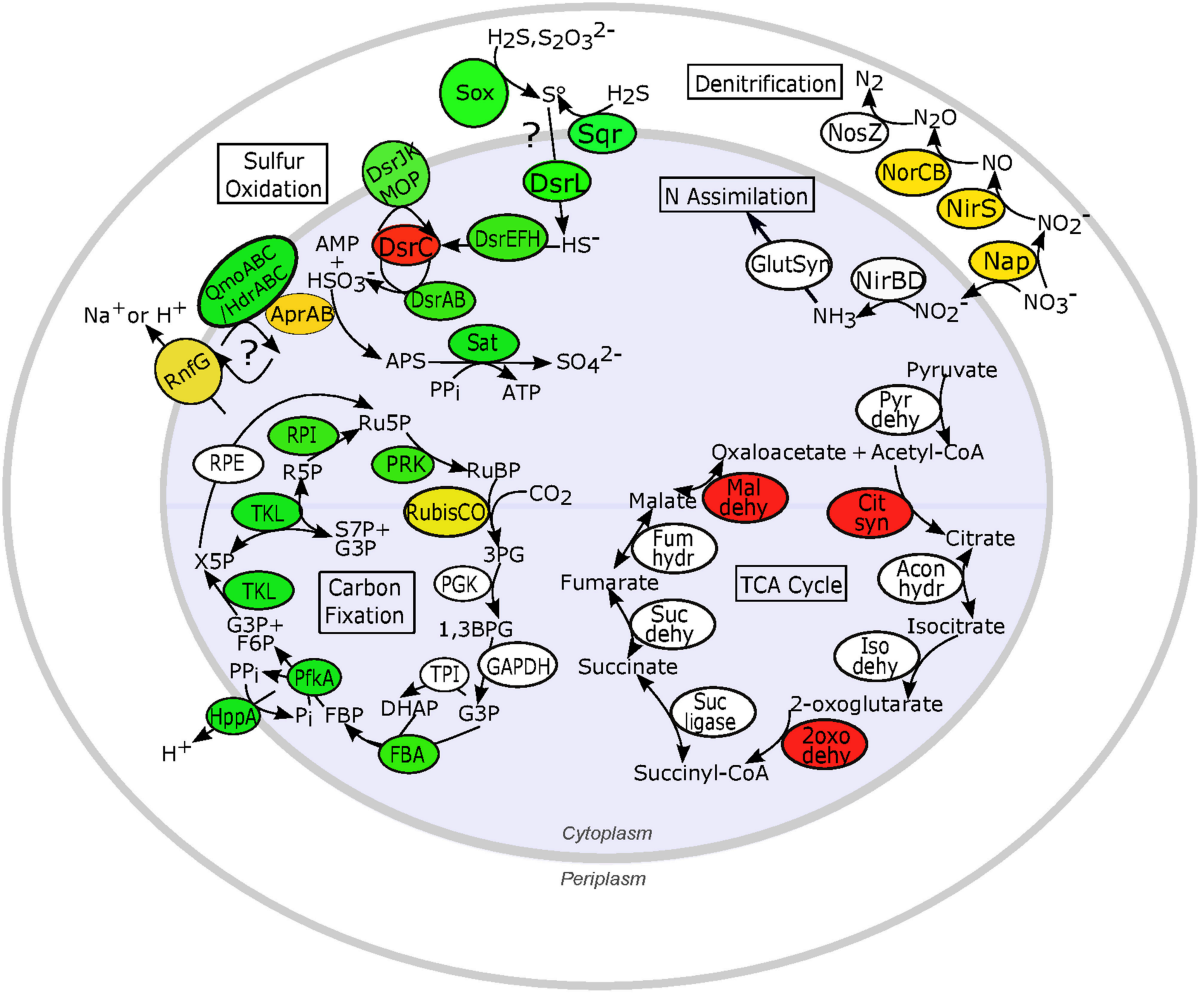
**Expression patterns for key proteins involved in proposed sulfur oxidation, nitrogen reduction and assimilation, and carbon metabolism pathways in chemosynthetic ***Ifremeria nautilei*** symbionts**. Proteins and complexes are colored based on transcript abundance in snail metatranscriptomes: green, constitutive expression (transcripts detected in all metatranscriptomes); yellow, HD expression > LD expression (FDR < 0.05); red, LD expression > HD expression (FDR < 0.05); white, sporadic or low expression (transcripts detected in fewer than 9 of the 12 metatranscriptomes); only proteins for which transcripts were detected are shown. Sulfur oxidation: Sox, Sox multi enzyme complex; AprAB, adenylylsulfate reductase; DsrL, Sulfur oxidation-associated protein DsrL; DsrEFH, putative sulfurtransferase complex; DsrC, putative bacterial heterodisulfide; DsrAB, reverse-type dissimilatory sulfite reductase; DsrJKMOP, sulfite reduction-associated complex DsrMKJOP; QmoABC/HdrABC, putative quinone-interacting membrane-bound oxidoreductase; RnfG, Rnf electron transport complex; Sat, sulfur adenylyltransferase; Sqr, sulfide quinone (oxido)reductase. N metabolism: Nap, periplasmic nitrate reductase; NirS, membrane-bound respiratory nitrite reductase; NirBD, NADH-dependent siroheme nitrite reductase; NorCB, nitric oxide reductase; NosZ, nitrous oxide reductase; GlutSyn, glutamine synthetase + glutamate synthase. TCA cycle: Pyr dehy, pyruvate dehydrogenase; Cit syn, citrate synthase; Acon hydr, Aconitate hydratase; Iso dehy, Isocitrate dehydrogenase; 2oxo dehy, 2-oxoglutarate dehydrogenase; Suc ligase, Succinyl-CoA ligase; Suc dehy, Succinate dehydrogenase; Fum hydr, Fumarate hydratase class I; Mal dehy, Malate dehydrogenase. Carbon fixation: RubisCO, Ribulose bisphosphate carboxylase; PGK, Phosphoglycerate kinase; GAPDH, NAD-dependent glyceraldehyde-3-phosphate dehydrogenase; TPI, Triosephosphate isomerase; FBA, Fructose bisphosphate aldolase; PfkA, Reversible pyrophosphate-dependent phosphofructokinase; HppA, membrane-bound proton-translocating pyrophosphatase; TKL, Transketolase; RPE, Ribulose-phosphate-3-epimerase; RPI, Ribose-5-phosphate isomerase A; PRK, Phosphoribulokinase; RuBP, Ribose-1,5-bisphosphate; 3PG, 3-phosphoglycerate; G3P, Glyceraldehyde-3-phosphate; DHAP, Dihydroxyacetone phosphate; FBP, Fructose-1,6-bisphosphate; F6P, Fructose-6-phosphate; X5P, Xylose-5-phosphate; Ru5P, Ribulose-5-phosphate; R5P, Ribose-5-phosphate; S7P, Sedoheptulose-7-phosphate; PPi, inorganic pyrophosphate; Pi, inorganic phosphate.

Sulfur oxidation genes typical of chemoautotrophic γ-proteobacteria were transcribed in all individuals (Figure 5). The canonical sulfur oxidation pathway in chemoautotrophic γ-proteobacteria is believed to start with the incomplete oxidation of sulfide or thiosulfate to elemental sulfur in the periplasm by the SoxABXYZ proteins (Ghosh and Dam, 2009). Through manual searches of BLASTX results (bit score > 50), we identified transcripts matching the s*oxABHWXYZ* genes (Table S4). Typical of γ-proteobacterial sulfur oxidizers, transcripts corresponding to the SoxCD proteins, which are necessary for complete oxidation of sulfide to sulfate by the Sox system, were not found, which may indicate that *I. nautilei* symbionts form elemental sulfur in the periplasm (Ghosh and Dam, 2009). SoxH, SoxK, and the thioredoxin SoxW were also detected in all metatranscriptomes and are commonly found in γ-proteobacterial sulfur oxidizers, but their roles in sulfide oxidation are unclear. In addition, manual searches of BLASTX results detected sulfide:quinone (oxido)reductase (Sqr) and sulfide dehydrogenase (Fcc) transcripts, both of which oxidize sulfide to polysulfides in the periplasm, although only Sqr has been shown to be necessary for sulfide oxidation (Dahl and Friedrich, 2008). The abundance of *sqr, fcc*, and s*oxABHWXYZ* transcripts was relatively uniform in all metatranscriptomes, demonstrating no differential expression based on metabolic activity or the concentration or type of electron donor (Tables S3, S4).

Following the production of elemental sulfur, sulfur-oxidizing γ-proteobacteria use the reverse dissimilatory sulfite reduction pathway (rDSR) to produce sulfite, followed by sulfite oxidation to sulfate by APS reductase (AprAB) and sulfate adenylytransferase (Sat; Dahl et al., 2005). Both MEGAN SEED protein classification and manual searches of BLASTX results showed that DSR genes *dsrABCEFHJKLMOPNR* were similarly represented in all metatranscriptomes (Tables S3, S4 and Figure 5). Transcription of *aprAB*, encoding the subunits of APS reductase, was correlated with sulfur metabolism rates (*R*^2^ = 0.99), and *aprAB* was the only sulfur metabolism gene identified by the baySeq model as significantly up-regulated in HD incubations (FDR = 0.006; Table S2). Multiple studies of free-living and endosymbiotic dissimilatory sulfur-metabolizing bacteria have found that AprAB expression is high in relation to other proteins (>6% of total proteome; Markert et al., 2007, 2011) and correlated with the energy state of the cell (Zhang et al., 2006; Pereira et al., 2008).

Transcription of the genes encoding the quinone-interacting membrane-bound oxidoreductase QmoABC complex, which is proposed to transfer electrons from AprAB to the periplasmic quinone pool, was sporadic among treatments (Figure 5). However, both SEED identifications and manual searches showed that all metatranscriptomes contained abundant sequences encoding heterodisulfide reductase HdrABC, which may be homologous to the QmoABC complex (Ramos et al., 2012). Indeed, genomic analysis of *Thiolapillus brandeum* strain Hiromi 1, a free-living strain closely related to *I. nautilei* sulfur-oxidizing symbionts, identified a chimeric cluster of *qmoAB-hdrBC* genes (Nunoura et al., 2014) and metagenomic/proteomic analysis of *Riftia pachyptila* γ-proteobacterial symbionts found abundant proteins annotated as heterodisulfide reductases with high similarity to *Chlorobium tepidum* QmoAB proteins (Markert et al., 2011). Thus, differentiation of these homologous protein complexes is problematic. In addition, the nature of electron transfer from AprAB to the periplasm is unclear and may involve heterodisulfide reductase and flavin-based electron bifurcation in order to conserve energy (Ramos et al., 2012). Evidence for expression of multiple proteins of the Rnf electron transport complex, which could couple with HdrABC to generate an ion motive force and thus ATP (Biegel et al., 2011; Ramos et al., 2012; Buckel and Thauer, 2013), was also observed in all symbiont metatranscriptomes (Figure 5 and Tables S3, S4), with transcripts for RnfG significantly more abundant in HD vs. LD incubations (FDR = 0.04; Table S2).

In summary, we observed expression of the full suite of expected sulfur oxidation genes in *I. nautilei* symbiont metatranscriptomes; however, expression of most sulfur oxidation genes, excluding AprAB, was not impacted by electron donor concentration or type. This result could be explained by several factors, including (i) stability and persistence of sulfur-oxidation transcripts produced prior to our experiments (i.e., during *in situ* activity), (ii) oxidation of elemental sulfur molecules stored by the symbionts to maintain thioautotrophic growth in the absence of sufficient exogenous reduced sulfur, or (iii) constitutive expression of most symbiont sulfur oxidation genes. Of these, the latter option may be most likely given that the predicted half-life of most mRNA molecules is considerably shorter than the duration of our experiments (Rauhut and Klug, 1999), and that there is no evidence that *I. nautilei* produce sulfur storage granules (Windoffer and Giere, 1997; Borowski et al., 2002). Rather, it has been suggested that indispensable metabolic pathways in bacteria are often constitutively expressed and are not repressible (Salmon et al., 2003, 2005). Thus, if *I. nautilei* symbionts are obligate sulfur oxidizers and cannot oxidize other electron donors, it is not surprising that sulfur oxidation genes are transcribed constitutively. Here, APS reductase (AprAB) and RnfG were the only sulfur oxidation genes whose transcription increased in response to reduced sulfur, suggesting that these proteins are tightly regulated by environmental conditions and are important for electron transport and energy conservation when either sulfide or thiosulfate is abundant. Indeed, other studies have also found that the transcription of AprAB is relatively responsive to environmental conditions compared to that of other sulfur oxidation genes (Zhang et al., 2006; Markert et al., 2007; Wendeberg et al., 2012). AprAB may therefore be a particularly good indicator of sulfur oxidation activity.

### Carbon metabolism

Previous work has shown that most γ-proteobacterial chemoautotrophic symbionts fix carbon via the Calvin-Benson-Bassham (CBB) cycle (Woyke et al., 2006; Duperron et al., 2007; Markert et al., 2007; Kleiner et al., 2012a; Sanders et al., 2013). Both SEED classification in MEGAN and manual searches of BLASTX data detected transcripts corresponding to all CBB cycle enzymes, although expression levels depended on the enzyme and incubation conditions (Figure 5 and Tables S3, S4). Furthermore, transcripts encoding the three key enzymes of the rTCA cycle (ATP citrate lyase, 2-oxoglutarate oxidoreductase, and fumarate reductase) were not detected, indicating that *I. nautilei* symbionts use only the CBB cycle for carbon fixation. Based on SEED classifications, form II Ribulose-1,5-bisphosphate carboxylase/oxygenase (RubisCO) was the most highly transcribed CBB cycle gene and the only CBB gene significantly more abundant in HD incubations (Table S2 and Figure 5). RubisCO transcript abundances and average ^13^C incorporation rates were both substantially higher in HD vs. LD incubations, although individual values were only moderately correlated (*R*^2^ = 0.24), suggesting that post-transcriptional regulation, redox poise, or other factors may control activity of the RubisCO enzyme and carbon incorporation rates (Beinart et al., 2015; Table 2). One individual in particular, snail HS3, exhibited a relative high abundance of RubisCO transcripts, but did not incorporate appreciable levels of ^13^C.

Form II RubisCO (CbbM), the only form detected in our data, is sensitive to oxygen and is hypothesized to function best in environments with low O_2_ and high CO_2_ concentrations (Tabita et al., 2008). However, it is thought that *cbbM* expression is unlikely to be directly regulated by CO_2_ concentration but may instead be regulated by redox conditions (Badger and Bek, 2008; Tabita et al., 2008; Alfreider et al., 2012). Our data are consistent with the hypothesis that cbbM transcription is induced when oxygen is low, and suppressed when oxygen concentrations are high, such as likely to be the case in the LD treatments if reduced access to electron donor decreases the symbionts' requirement for oxygen. Thus, transcription levels may be tightly linked to redox conditions experienced by the symbionts. Further investigation on the regulation of RubisCO in symbionts is needed to understand how this critical enzyme is regulated and optimized for conditions within the host.

Transcripts matching phosphoribulokinase, fructose-bisphosphate aldolase, transketolase, and ribulose-5-phosphate isomerase were detected in all 12 metatranscriptomes, whereas transcripts for other CBB cycle enzymes were detected at lower levels and more sporadically among datasets (Table S3). As has been reported for symbionts of deep-sea tubeworms (*Riftia pachyptila*), clams (*Calyptogena magnifica*), and shallow water gutless marine worms, we did not detect transcription of the genes encoding sedoheptulose-1,7-bisphosphatase and fructose-1,6-bisphosphatase, which are necessary to regenerate ribulose-1,5-bisphosphate (Markert et al., 2007; Newton et al., 2007; Kleiner et al., 2012b). However, manual searches of BLASTX results detected transcription of genes for reversible pyrophosphate-dependent phosphofructokinase (PfkA) and a membrane-bound proton-translocating pyrophosphatase (HppA), which together have been proposed to replace sedoheptulose-1,7-bisphosphatase and fructose-1,6-bisphosphatase in some chemolithotrophic symbionts (Table S4). Using reversible PfkA in combination with HppA in the CBB cycle reduces the net ATP consumption of carbon fixation as the membrane-bound proton-translocating pyrophosphatase establishes a proton gradient across the cytoplasmic membrane, allowing for ATP generation by ATP synthase (Reshetnikov et al., 2008; Kleiner et al., 2012a,b). In support of this hypothesis, PfkA enzyme activity was detected in strain Hiromi 1, a strain closely related to *I. nautilei* sulfur-oxidizing symbionts, *pfkA* is conserved in all complete genomes of sulfur-oxidizing γ-proteobacteria available in public databases, and *pfkA:hppA* are co-localized in the genomes of many free-living and symbiotic chemoautotrophic bacteria (Kleiner et al., 2012a,b; Nunoura et al., 2014).

Evidence for expression of bacterial oxidative TCA enzymes was observed in all metatranscriptomes, and transcripts corresponding to citrate synthase, 2-oxoglutarate dehydrogenase, and malate dehydrogenase were significantly more abundant in LD vs. HD treatments based on the baySeq differential expression analysis (Table S2). In addition, transcripts encoding two enzymes involved in the biosynthesis of lipoyl cofactors (octanoate-[acyl-carrier-protein]-protein-N-octanoyltransferase and lipoate synthase) essential for the activation of oxidative TCA cycle enzymes and a long chain fatty acid coenzyme A ligase involved in activation of fatty acid breakdown were also significantly enriched in LD metatranscriptomes (Table S2).

Activation of fatty acids and expression of oxidative TCA cycle enzymes when electron donors are limiting could provide a number of advantages for symbiont survival. Incorporation of fatty acids and other organic carbon through the TCA cycle is less energy intensive than fixing inorganic carbon and also produces NADPH, which presumably would be in short supply when electron donors are not available (Nunoura et al., 2014). However, net heterotrophic growth requires uptake of organic compounds, and we did not find evidence for the expression of genes involved in the transport of organic compounds into the cell (Kleiner et al., 2012b). Alternatively, increased expression of TCA cycle enzymes could be driven by the oxidizing conditions in the LD incubations, as has been observed in multiple studies in *Escherichia coli* (Salmon et al., 2003, 2005; Shalel-Levanon et al., 2005; Toya et al., 2012).

It is not clear from our data whether *I. nautilei* symbionts are performing net heterotrophy or are using internal carbon molecules such as fatty acids and glycogen as a source of carbon and reducing equivalents when electron donors are limited. Free-living strains related to *I. nautilei* symbionts have been observed to incorporate complex organic carbon sources such as fumarate, formate, citrate, pyruvate, and peptone (Sievert and Vetriani, 2012; Nunoura et al., 2014). Our data raise the possibility that *I. nautilei* symbionts are mixotrophic, fixing inorganic carbon when reduced sulfur is available, but switching to chemoheterotrophy when energy supplies are limiting.

### Nitrogen metabolism

Inorganic nitrogen compounds are generally abundant at hydrothermal vents, while organic nitrogen compounds are scarce (Johnson et al., 1986). Vent fluids may contain ammonium, which can be assimilated by many microorganisms. However, nitrate is typically more abundant and can be used as both an oxidant for energy generation (respiration) and a primary nitrogen source for assimilation. Both processes begin with nitrate reduction to nitrite, but proceed through different enzymatic pathways (Potter et al., 1999; Klotz and Stein, 2008). The next step in nitrogen assimilation is the cytoplasmic reduction of nitrite to ammonia by the complex NirBD and assimilation by glutamine synthetase/glutamate synthase or asparagine synthase. In the dissimilatory pathway, nitrite may be further reduced to nitrogen gas (denitrification) in three steps: nitrite reduction by NirS or NirK, nitric oxide reduction by the NorCB complex, and finally nitrous oxide reduction by NosZ. Alternatively, nitrite can be reduced to ammonium by Nrf to generate energy in a process of dissimilatory nitrate reduction to ammonia (Potter et al., 1999; Klotz and Stein, 2008).

Metatranscriptomes from HD incubations were significantly enriched in transcripts encoding proteins of both assimilatory and dissimilatory (respiratory) nitrate reduction and ammonium assimilation compared to LD treatments (Figure 5 and Table S2). Interestingly, no transcripts corresponding to the membrane-bound dissimilatory Nar or cytoplasmic assimilatory Nas nitrate reductases were identified. We only found transcripts encoding the periplasmic nitrate reductase NapCFGH proteins, which may be used in either assimilation or respiration, and in some cases may serve as an electron shunt when electron flow through the aerobic respiratory chain is restricted due to low oxygen concentrations (Potter et al., 1999; Klotz and Stein, 2008). The relative abundance of Nap transcripts was on average over 20X higher in HD compared to LD conditions (Table S3). Transcripts encoding a complete suite of respiratory denitrification [*nirS, norCB*, and *nosZ*] and cytoplasmic nitrogen assimilation [nirBD, glutamine synthetase-glutamate synthase] enzymes were also significantly enriched in HD metatranscriptomes (Table S2). These results suggest that when sufficient electron donor is available *I. nautilei* symbionts reduce nitrate to nitrite in the periplasm and subsequently use it for respiration (denitrification) in the periplasm or transport the nitrite to the cytoplasm for assimilation (Figure 5). Metatranscriptomic analysis of sulfur-oxidizing symbionts of the vent snail *Alviniconcha* found evidence for the same nitrate assimilation and denitrification pathways reported here (Sanders et al., 2013). Furthermore, a recent study showed that a close free-living relative of the *I. nautilei* symbiont coupled complete denitrification to sulfur oxidation and reached higher cell densities with nitrate as electron acceptor compared to oxygen (Nunoura et al., 2014).

Using the periplasmic Nap complex for nitrate reduction provides considerable adaptability in nitrogen metabolism and respiration in response to dynamic nitrogen concentrations and redox conditions. After reduction of nitrate to nitrite in the periplasm, nitrite can be transported into the cytoplasm only when it is needed for assimilation, keeping cytoplasmic nitrite concentrations low and thus avoiding nitrite toxicity (Rowe et al., 1979). Additionally, unlike the respiratory Nar proteins, the Nap complex is not inhibited by oxygen (Potter et al., 1999). Consequently, the Nap complex can be expressed under aerobic conditions, potentially allowing symbionts to use nitrate as an electron acceptor and avoid competition with the host for oxygen (Sanders et al., 2013). Our results confirm that sulfur-oxidizing symbionts tightly regulate the expression of nitrogen assimilation and denitrification genes in response to reduced sulfur and oxygen availability.

### Stress response

Symbiont populations in LD incubations express genes that are consistent with increased oxidative stress (Chen et al., 2009). Of 19 protein-coding genes identified as significantly more abundant in LD incubations using the baySeq model, 10 were related to the bacterial stress response (Table S2). In bacteria, a highly conserved “heat-shock” stress response occurs in response to heat, starvation, radiation, and oxidative agents (Neidhardt et al., 1984). The heat-shock response involves production of a set of heat-shock proteins, many of which are molecular chaperones involved in transit across membranes, targeted proteolysis, and polypeptide folding (Genevaux et al., 2007). Stress-induced transcription of heat-shock genes is typically controlled by binding of the sigma factor RpoH to RNA polymerase (Bukau, 1993). Based on SEED protein classification in MEGAN, metatranscriptomes from LD incubations contained 9X more RpoH-related transcripts than those from HD incubations, indicating that the heat-shock response was induced in symbionts without sufficient electron donor in the presence of oxygen (Table S2).

In accordance with an increase in RpoH, transcripts encoding proteins in the DnaK chaperone system and the Clp/Hsp100 family of ATP-dependent protein remodeling machines also increased in LD incubations, notably representing the most abundant transcripts in ND datasets. The DnaK chaperone system is composed of DnaK, DnaJ, and GrpE, which work together with Clp/Hsp100 family proteins to catalyze protein disaggregation and refolding or protein degradation during physiologic stress (Dougan et al., 2002). Here, DnaK, DnaJ, and GrpE transcripts were significantly enriched in LD incubations, although DnaJ and GrpE transcripts were 10 to 100X less abundant than DnaK (Tables S2, S3). Transcripts encoding ClpA and ClpX, as well as FtsH subunits and peptidases associated with ATP-dependent protein degradation (Dougan et al., 2002), were also significantly enriched under LD conditions (Table S2). Other subunits associated with the Clp/Hsp100 family, such as ClpB and ClpS, were highly expressed in LD incubations, but were not significantly differentially expressed under the criteria of the baySeq model (Table S3).

Transcription of genes involved in iron regulation and 4Fe-4S cluster biosynthesis were also significantly more abundant under LD conditions, potentially due to the importance of iron homeostasis for cell survival during oxidative stress (Table S2). Ferrous iron reacts with the hydrogen peroxide present during oxidative stress to form damaging peroxide radicals. The rate of oxidative damage is therefore elevated when cytoplasmic iron concentrations are high (Imlay, 2013). Transcripts related to the MerR family of metalloregulators, which detect and respond to reactive metals and changes in redox conditions, were significantly enriched in LD metatranscriptomes. These included transcripts encoding the oxidative stress metalloregulator SoxR, which is known to induce expression of multiple proteins that mitigate cellular damage, including superoxide dismutase and the regulatory Fur protein (Brown et al., 2003; Imlay, 2013). Both the Fur protein, which suppresses iron import and utilization, and superoxide dismutase, were significantly more abundant in LD incubations (Table S2), suggesting that *I. nautilei* symbionts without sufficient electron donating compounds were attempting to limit iron uptake and neutralize superoxide to avoid oxidative damage.

Proteins containing 4Fe-4S centers and mononuclear iron also are very susceptible to damage from reactive oxygen species (Imlay, 2013). Transcripts from two Fe-S cluster assembly pathways, the Isc (iron sulfur cluster) system and the Suf (sulfur formation) system, were detected in all snail metatranscriptomes (Table S3). The Suf system has been found to operate specifically to protect and assemble Fe-S clusters during oxidative stress (Ayala-Castro et al., 2008). In accordance with this suggested function, transcripts encoding a key scaffold protein (SufB) in the Suf system were on average 20X more abundant in LD vs. HD incubations (Table S2). LD transcriptomes were also significantly enriched in transcripts for the DNA repair enzyme RecA (Imlay, 2013) and the stress-induced morphogene BolA (Santos et al., 1999). Together, these results indicate significant oxidative stress in symbiont populations without sufficient electron donor, suggesting that *I. nautilei* cannot shield its symbionts from fluctuations in redox conditions that may negatively impact symbiont survival.

### Methane (C1) metabolism

Despite a presumed lack of exogenous methane in these experiments, we detected transcripts indicating that methanotroph-related *I. nautilei* symbionts were active during the incubations. Phylogenetic analysis of 16S rRNA transcripts and amplified rRNA genes placed the methanotroph-related *I. nautilei* symbionts in a monophyletic group within the γ-proteobacterial family *Methylococcaceae* (Figure 2)*. Methylococcaceae* are type I methanotrophs typically characterized by the use of the ribulose monophosphate (RuMP) pathway for incorporation of C1 carbon into biomass and a membrane-associated particulate methane monooxygenase (pMMO) that catalyzes the oxidation of methane to methanol (Chistoserdova, 2011). Manual searches of BLASTX files revealed transcripts matching the pMMO genes *pmoCAB*, accounting for 0.1-1.5% of non-rRNA reads in the TS, LS, and ND datasets (Table S4). Recovered pMMO sequences shared >85% amino acid similarity to pMMO proteins from *Methylomicrobium album, Methylomicrobium buryatense*, and *Methylomarinum vadi*, supporting the phylogenetic placement of these symbionts within the *Methylococcaceae*, but not specifically related to methanotrophic symbionts of deep-sea bathymodiolin mussels. No transcripts indicative of sMMO were detected (Hanson and Hanson, 1996; Chistoserdova, 2011). PmoC-related reads represented the most abundant methanotroph-related functional gene transcript observed in the TS, LS, and ND metatranscriptomes, and were 5–10X more abundant than PmoA or PmoB transcripts. Two previous transcriptomic studies of alpha-proteobacterial methanotroph pure cultures observed that the *pmoCAB* operon is the most highly expressed operon in the genome when sufficient copper is present, with *pmoC* gene expression 6–7X higher than *pmoA/B* (Matsen et al., 2013; Vorobev et al., 2014).

Evidence for the further oxidation and assimilation of methane was also detected, although transcripts from these pathways were one to two orders of magnitude less abundant than pMMO-related transcripts (Table S4). Transcripts encoding methanol dehydrogenase proteins (MDH), which catalyze the oxidation of methanol to formaldehyde (Chistoserdova, 2011), were present in three of the nine methanotroph-containing metatranscriptomes. In addition, six metatranscriptomes contained transcripts matching methanotroph-related genes for the biosynthesis of the methanol dehydrogenase cofactor pyrroloquinoline quinone. Expression of unique genes for formaldehyde oxidation and assimilation through the RuMP pathway (3-hexulose-6-phosphate synthase and 3-hexulose-6-phosphate isomerase) were also detected in six of the nine methanotroph-containing metatranscriptomes (Table S4).

Collectively, these results confirm the transcriptional activity of a methanotrophy pathway in *I. nautilei* symbionts. The presence of methanotrophy-related transcripts despite a lack of C1 compounds in these experiments is surprising as no methane was introduced into these experiments, and the chance of methane being present in any of the injected mixed gases is very low. Our data may therefore be explained by a constitutive transcription of methanotrophy genes, or a relatively high stability of methanotrophy transcripts. Indeed, both constitutive expression and high transcript stability have been reported for the *pmoCAB* operon (Lieberman and Rosenzweig, 2004; Deana and Belasco, 2005; Chen et al., 2008; Wendeberg et al., 2012; Matsen et al., 2013). Alternatively, putatively methanotrophic *I. nautilei* symbionts could be growing on substrates other than methane, such as methylated sulfur compounds or even multi-carbon substrates. However, this explanation seems unlikely, as all *Methylococcaceae* characterized to date are obligate methanotrophs (Chistoserdova, 2011). The functional contributions of *I. nautilei* methanotrophs to holobiont fitness remain to be determined.

## Concluding remarks

This study of deep-sea vent snails is unique in that it examined the transcriptional responses of bacterial symbionts under well-controlled environmental conditions. These results show that while *I. nautilei* symbionts can use either sulfide or thiosulfate for energy during chemoautotrophic growth (as observed in Beinart et al., 2015), the transition between these substrates appears to have surprisingly little effect on symbiont transcription patterns. Periplasmic nitrate reduction appears critical at higher sulfide or thiosulfate conditions, likely reflecting a dependence on nitrate respiration at elevated sulfur species concentrations, as well as enhanced nitrogen assimilation to meet potentially elevated growth rates.

Notably, when sulfide is low or absent, there are marked and unexpected changes in symbiont transcription. First, *I. nautilei* symbionts express genes that are consistent with mixotrophy, suggesting the possibility of transitioning to heterotrophic growth when energy for CO_2_ fixation is limiting. The origin and composition of the organic carbon for symbiont heterotrophy is unknown in *I. nautilei*, but could involve recycling of organics leaked from host cells or the catabolism of internally stored carbon (Kleiner et al., 2012b). Second, these data suggest that at lower sulfide concentrations *I. nautilei* symbionts may experience oxidative stress. We posit that the symbionts may be experiencing a reduction in reactive oxygen species scavenging when electron donors are scarce, or alternatively may be exposed to elevated reactive oxygen species concentrations caused by stressed hosts. While the causal factor remains unclear, it is important to note that these conditions are comparable to those found *in situ*, especially around lower flow diffuse vents. Third, a striking commonality among many of the differentially expressed genes identified in this study, including form II RubisCO, oxidative TCA cycle enzymes, and genes involved in iron uptake, is that redox potential is implicated in their regulation based on studies in other bacteria; thus environmental redox conditions may control many processes in *I. nautilei* symbionts.

Our results also highlight the variability of *I. nautilei* symbiont community composition across the Lau Basin vent system and provide the first molecular evidence for the presence and activity of methanotrophic symbionts in *Ifremeria*. The persistence of methanotrophy transcripts in our incubations, despite the lack of a clear methane source, may reflect an adaptation to the dynamic vent environment, with the *Ifremeria*-associated bacterial community being poised to rapidly consume methane when this energy source becomes available.

Together these data reveal that vent geochemical dynamics affect a broad range of metabolic systems and subsystems, such as carbon, nitrogen and sulfur metabolism. Such variation will in turn govern the extent to which the holobiont's (host + symbiont) activity influences environmental geochemistry. Finally, the apparent response to low or no sulfide was quite striking, and future studies should further examine the extent to which oxidative stress influences symbiont function *in situ*. It should be noted that the relative abundances of transcripts, for example those encoding RubisCO, AprAB, or stress proteins, may be reasonably accurate indicators of substrate conditions and symbiont metabolic state. Other loci, however, appear less transcriptionally responsive to environmental change and their expression patterns should be interpreted with caution. In the future, similar controlled experiments that couple measurements of chemical flux (e.g., carbon fixation, sulfur and methane oxidation) to biomolecule abundances are necessary for determining which molecular patterns accurately reflect symbiont physiology, and therefore the extent to which meta-omic datasets can be used for predictive models of symbiont contributions to holobiont fitness.

## Author contributions

RB and PG conceived and designed the study, performed on-board experiments, and extracted nucleic acids. SS and FS designed the sequencing and analysis approach. SS, NS, SG, and AS performed sequencing reactions and sequence processing. SS, NS, SG, and PR analyzed the data. SS and RB wrote the paper with contributions from FS, PG, and NS.

### Conflict of interest statement

The authors declare that the research was conducted in the absence of any commercial or financial relationships that could be construed as a potential conflict of interest.

## Supplementary material

Figure S1 **Maps showing the vent fields ABE and Tu'i Malila at the Eastern Lau Spreading Center where individual snails used in the experimental treatments were collected**.

Table S1 **List of differentially expressed SEED Level 1 subsystems including normalized transcript abundance and false discovery rate (FDR) values**.

Table S2 **List of differentially expressed SEED Level functional proteins as annotated in MEGAN5 including normalized transcript abundance and false discovery rate (FDR) values**.

Table S3 **Normalized abundance of transcripts in all 12 metatranscriptomes corresponding to SEED subsystem functional proteins annotated in MEGAN5**.

Table S4 **Abundance of specific transcripts of interest (see Materials and Methods) in all 12 metatranscriptomes from manual searches of processed BLASTX results (bit score >50) normalized to 100 000 non-rRNA reads per metatranscriptome**.
