# Supplementary material for: Metatranscriptional Response of Chemoautotrophic Ifremeria nautilei Endosymbionts to Differing Sulfur Regimes
Source: Front Microbiol. 2016 Jul 19;7:1074. doi: 10.3389/fmicb.2016.01074 (PMC4949241; doi:10.3389/fmicb.2016.01074)
Supplement: Figure S1 — Maps showing the vent fields ABE and Tu'i Malila at the Eastern Lau Spreading Center where individual snails used in the experimental treatments were collected. [file Image1.PDF]

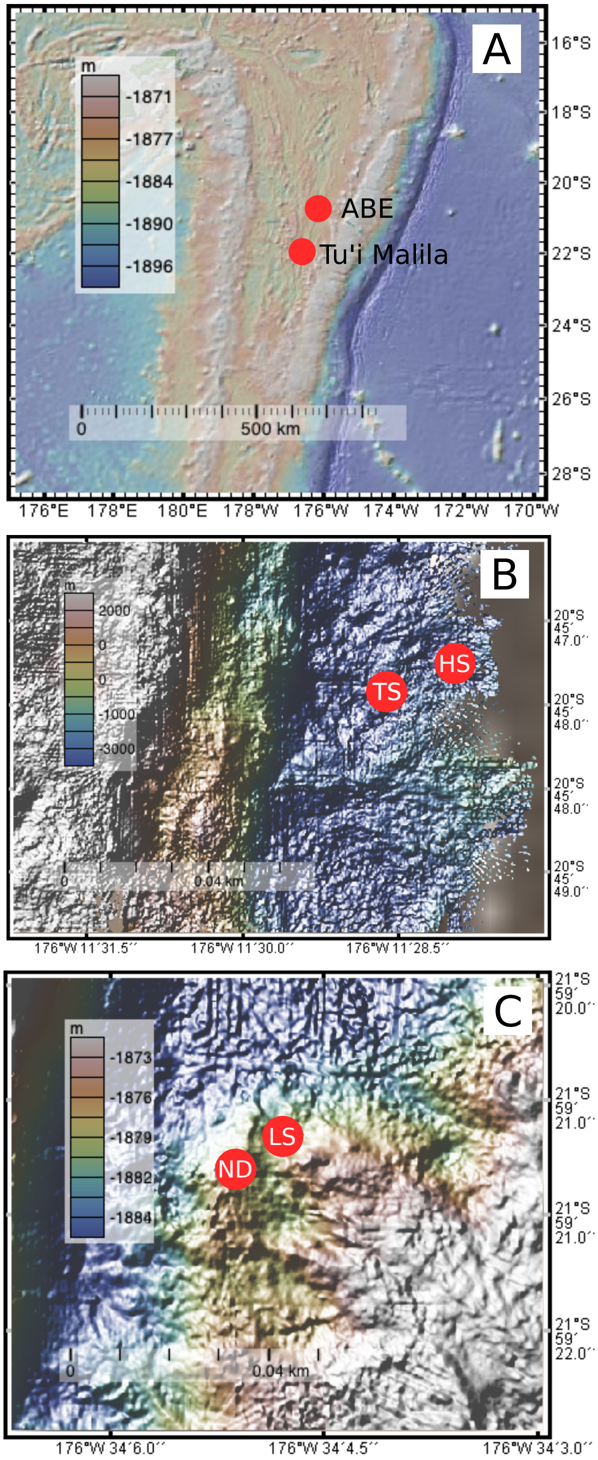

Figure S1: Maps showing the vent fields ABE and Tu'i Malila (A) at the Eastern Lau Spreading Center, as well as the sites within ABE where individuals used in the high sulfide (HS) and thiosulfate (TS) treatments were collected (B); and the sites within Tu'i Malila where individuals used in the low sulfide (LS) and no donor (ND) treatments were collected (C). Maps were created with GeoMapApp (http://www.geomapapp.org), using the TUIM05MV:Jason II SM2000 – 25 cm bathymetry grids.
